# Influence of *Lactobacillus helveticus* ZF22 and TR1-1-3 strains on the aromatic flavor of fermented sausages

**DOI:** 10.3389/fnut.2022.1058109

**Published:** 2023-01-09

**Authors:** Jianjun Tian, Xueqian Yang, Kaiping Zhang, Yanhong Zhao, Feng Cheng, Ye Jin

**Affiliations:** ^1^College of Food Science and Engineering, Inner Mongolia Agricultural University, Hohhot, China; ^2^Department of Cooking & Food Processing, Inner Mongolia Business & Trade Vocational College, Hohhot, China

**Keywords:** *Lactobacillus helveticus*, fermented sausage, volatile compounds, salt tolerance, acid tolerance, nitrite tolerance, antibacterial

## Abstract

In this study, five strains isolated from traditional Inner Mongolian air-dried meat products were used, two *Lactobacillus helveticus* strains, ZF22 and TR1-1-3, with potent antibacterial activity, acid, salt, and nitrite tolerance, were selected for this study. Lactic acid bacteria (LAB) (*Lactobacillus helveticus* ZF22 and TR1-1-3) were inoculated into fermented sausages at 10^7^ CFU/g and their volatiles were studied during fermentation and storage. Clustering heat map and principal component analysis (PCA) were used to identify differentiating flavor components in uninoculated and inoculated sausages. The results showed that 72 volatile flavor substances were identified during the fermentation of the fermented sausages and that inoculation with *Lactobacillus helveticus* ZF22 and TR1-1-3 increased the proportion of acids, ketones and alkanes. Moreover, the clustering heat map demonstrated that esters such as ethyl isobutyrate, ethyl acetate, and ethyl valerate were more abundant in TR1-1-3 and ZF22 than ZR. The PCA analysis showed that the volatile compounds of the three fermented sausages were distributed in separate quadrants, suggesting that the volatile compound compositions of the three fermented sausages differed significantly. Our findings suggest that inoculating fermented sausages with *Lactobacillus helveticus* TR1-1-3 and ZF22 can improve flavor by enhancing the type and amount of flavor compounds.

## 1. Introduction

Two forms of fermentation are present in fermented sausages: natural fermentation and guided fermentation with starter cultures (1). Due to the lack of controllability of naturally fermented sausages, the empirical method is used for spontaneous fermentation (1). It is challenging to ensure the final product’s taste, but introducing a starter can shorten production time while ensuring product safety and quality (2). The addition of starter cultures can reduce the ripening period of the meat while also improving color, taste, and safety (3, 4). The starter cultures are increasingly used in modern sausages production to control product performance, such as flavor and color consistency (5).

Lactic acid bacteria (LAB), which are widely utilized as starter cultures in fermented meat products, are facultative anaerobic bacteria. *Lactobacillus*, specifically *Latilactobacillus sakei*, *Latilactobacillus curvatus*, and *Lactiplantibacillus pentosus*, are the primarily utilized species (6, 7). LAB are responsible for the quick fermentation of additional carbohydrates in fermented meat products, resulting in a pH decrease (8). Visessanguan et al. (9) reported that *Lactiplantibacillus plantarum*, *Latilactobacillus sakei*, and *Lactiplantibacillus pentosus* were prominent in meat fermentation.

With the advancement of manufacturing technology and equipment in recent years, research on fermented meat products’ flavor, nutritional qualities, and safety has become a widespread issue (10). The flavor is an essential component of the edible quality of meat and meat products (11). The scent and flavor of fermented meat rely on volatile molecules (12). *Lactobacillus lactis*, *Lactiplantibacillus plantarum*, *Companilactobacillus alimentarius*, *Latilactobacillus sakei*, and *Weissella hellenica* all help to produce significant volatile compounds (13). *Lactiplantibacillus plantarum* and *Staphylococcus xylosus* were reported to suppress the growth of pathogenic bacteria, reduce rancidity, and improve the flavor of fermented sausages by Xiao et al. (14). Gas chromatography-mass spectrometry (GC/MS) is the most commonly used method for assessing the olfactory features of meat products because it provides a dependable instrument for the qualitative and quantitative examination of volatile and non-volatile compounds (15). SPME-GC/MS has been frequently employed in volatile composition investigations in meat products such as fermented sausages and dry-cured ham (16, 17). However, little research has investigated the taste features of fermented meat products using GC/MS in conjunction with primary component analysis.

The primary objective of this study was to select LAB with high fermentation capabilities from five strains of LAB and use them as the fermentation agent in fermented sausages. Solid phase microextraction-GC/MS was used to study changes in volatile flavor components in fermented sausages. The volatile flavor cluster heat map was created, and the specific volatiles in the fermented sausages fermentation process were analyzed using principal component analysis (PCA).

## 2. Materials and methods

### 2.1. Materials and chemicals

Mutton back fat and lean mutton (center temperature of 4°C) were obtained from Inner Mongolia Grassland Jingxin Food Co. (Bayan Nur, China) less than 24 h after slaughtering and transported on ice to the Inner Mongolia Agricultural University’s meat science laboratory. 2-methyl-3-heptanone was purchased from Sigma Aldrich (USA), sodium nitrite and concentrated hydrochloric acid from Sinopill Chemical Reagents (Co., Ltd. China), and TPY medium from Beijing AOBOXing Biotechnology (Co., Ltd. Beijing China). All chemicals and solvents were of analytical grade.

### 2.2. Bacterial cultures and culture media

This study used five LAB strains (*Lacticaseibacillus casei* ZF6, *Lacticaseibacillus casei* ZF8, *Lactobacillus helveticus* ZF22, *Lactobacillus helveticus* ZF13, and *Lactobacillus helveticus* TR1-1-3), including strains previously obtained from traditional air-dried meat products in Inner Mongolia. The pathogenic bacteria used in these inhibition tests included *Escherichia coli* (CICC10389), *Bacillus subtilis* (ATCC6633), *Staphylococcus aureus* (CICC10384); obtained from the China Center of Industrial Culture Collection, and *Salmonella* (BNCC358231) purchased from BeNa Culture Collection (China). LAB was cultured in Trypticase-Phytone-Yeast medium (TPY), and TPY broth was prepared following the Havas et al. (18) described method. The TPY broth contain (per liter), Trypton (10 g), phytone (5 g), glucose (5 g), yeast extract (2.5 g), Tween 80 (1 ml), L-cysteine (0.5 g), K_2_HPO_4_ (2 g), MgCl_2_*6H_2_O (0.25 g), CaCl_2_ (0.15 g), and FeCl_3_ (0.03 g) with a pH 6.0 ± 0.2. The pathogenic bacteria were cultured in a Luria-Bertani (LB) medium according to Sezonov et al. (19) protocol that comprised Tryptone (10 g/L); Yeast extract (5 g/L); and NaCl (10 g/L) at a final pH of 7 ± 0.2.

### 2.3. Determination of salt tolerance

Trypticase-Phytone-Yeast liquid medium was inoculated with five strains (*Lacticaseibacillus casei* ZF6, *Lacticaseibacillus casei* ZF8, *Lactobacillus helveticus* ZF13, *Lactobacillus helveticus* ZF22, and *Lactobacillus helveticus* TR1-1-3) of LAB preserved in glycerol for two activations in TPY medium. The strains were collected by centrifugation (3000 × rpm, 10 min, 4°C), suspended in 0.9% (w/v) NaCl, and diluted to a final OD_600_ nm of 0.5. Diluted cultures were then inoculated at 2% (v/v) into 10 mL of TPY medium with different NaCl concentration (3, 6, 7.5, 9, 12%) (20, 21). Five strains were cultured in TPY medium with a NaCl concentration of 0 as a control group. The absorbance at OD_600_ nm was measured after 24 h of incubation.

### 2.4. Determination of acid tolerance

The strains were cultured and processed in the same manner as in experiment 2.3, and then the diluted cultures were inoculated at 2% (v/v) into 10 mL of TPY medium at various pH (3.5, 4.5, 5.5, 6.5) (22). Five strains of LAB were cultured in the original TPY medium as a control. After 24 h of incubation, the absorbance at OD_600_ nm was recorded.

### 2.5. Determination of nitrite tolerance

The strains were cultured and pre-treated as in experiment 2.3, and then diluted cultures were inoculated at 2% (v/v) into 10 mL of TPY medium at different nitrite concentrations (50, 100, 150, 200, and 300 mg/kg) (23). Five strains were cultured in the original TPY medium as a control group. The absorbance at OD_600_ nm was measured after 24 h of incubation.

### 2.6. Bacteriostatic characteristics of the strain

Four pathogenic bacteria (*Salmonella* BNCC358231, *Escherichia coli* CICC10389, *Staphylococcus aureus* CICC10384, and *Bacillus subtilis* ATCC6633) were inoculated in LB liquid medium and subjected to two rounds of activation (37°C, 24 h). The pathogenic bacteria liquid utilized in the bacteriostatic test serves as the second activation of the pathogenic bacteria. Five lactic acid bacterial strains were tested for antibacterial activity using the Oxford cup double agar diffusion method. Oxford cups were put on top of sterile water agar, poured into the Petri dish bottoms, and left to dry. A 100 μL indicator bacteria containing 10^7^ CFU/mL was poured into the culture dish in the Oxford cup, along with TPY solid medium. The Oxford cup was removed after drying, and 200 μL of LAB test solution was poured into the wells. Three parallel experiments were carried out in each group after the culture plate was placed in an incubator for 24 h at 37°C. The observation findings were removed, and the inhibitory zone’s diameter was measured using an electronic digital caliper (24).

### 2.7. Preparation of fermented sausages

Sausages were made with lean mutton (700 g) and mutton back fat (200 g) minced through a 1.5 cm plate, along with the following additives (g/kg meat): sodium chloride (25), sucrose (5.0), glucose (5.0), sodium nitrite (0.1), pepper (5.0), and Baijiu (20). Strain cell pellets were diluted in 0.9% normal saline to create cell suspension. The meat, fat and seasoning are put in a food mixer and mixed well. Then, the meat batter was inoculated with the cell suspension at a concentration of 10^7^ CFU/g meat. The treatment group uses starters, while the control group does not, and both groups adhere to the same protocols. The mixed lamb filling is dried at 4°C for 24 h, formed into sausages, and then moved to an incubator where it is fermented for 72 h at 25°C and 95% relative humidity. At the end of fermentation, the sausages are dried in a constant temperature oven at 15°C and 70–75% relative humidity for 4 days. After drying, the fermented sausages are ripened in a constant humidity chamber at 10°C for 3 days with a relative humidity of 60–65%. The matured fermented sausages are stored at 4°C for 15–30 days.

Three groups of the fermented sausages were developed: a control group (ZR) with no additional fermentative; a group (TR1-1-3) with 10^7^ CFU/g *L. helveticus* TR1-1-3; and a group (ZF22) with 10^7^ CFU/g *L. helveticus* ZF22.

### 2.8. Measurement of pH and bacterial counts

The pH of fermented sausages was measured according to the method described by Chen et al. (26).

An aliquot (10.0 g) of minced sausages was transferred aseptically to a sterile plastic bag and mashed in 90.0 mL of isotonic saline in a gastroscope for 1 min according to the method of Hu et al. (13). Samples were appropriately decimal diluted using isotonic saline and 0.1 mL of each dilution was plated in triplicate on agar. After incubation at 37°C for 48 h, LAB populations were counted on MRS agar.

### 2.9. Determination of volatile flavor

Determination of volatile flavor components of fermented sausages by GC–MS according to Wen et al. (25). The casing was taken off the fermented lamb sausages at different processing stages. It was then chopped, weighed to 5 g, put in a 20 mL sample vial, and 1 μL of the standard was added. To expose the quartz fiber tip to the sample’s upper space, the aging extraction head was placed into the vial and was withdrawn after 40 min at 60°C. The extraction head was desorbed at the GC inlet (250°C) for 3 min. Conditions of determination: DB–5 columns (30 m × 0.25 mm × 0.25 μm), the carrier gas with the flow rate of 1.0 mL/min, the temperature of sample introduction and the interface is 250°C, temperature rising procedure with an initial temperature of 40°C, then holding for 3 min, raising the temperature from 4°C/min to 150°C, holding for 1 min, then raising the temperature from 5°C/min to 200°C, holding for 5 min, unsplit stream sampling. The ion source and transmission line temperatures are 250°C, and the quality scanning range is 30–400 m/z. A 1-min solvent delay was established. Searches with MEANLIB, NISTDEMO, and the Wiley Library were used to characterize the mass spectrometry data, and a match of >800 served as the basis for confirmation.

### 2.10. Statistical analysis

The experimental data were statistically analyzed using IBM SPSS Statistics 23.0 (IBM, Chicago, IL, USA). The outcomes were statistically significant using a one-way analysis of variance (ANOVA) and Duncan’s multi-range test with a significance level of *p* < 0.05. Each experiment’s results were reported in triplicate along with their mean ± SD (standard deviation). The PCA plugin and the Heat Map with the Dendrogram plugin in Origin 2021 were utilized for PCA and cluster heat maps, respectively.

## 3. Results and discussion

### 3.1. Lactic acid bacteria growth at different salt concentrations

Salt is an auxiliary material in the fermented mutton sausages used for seasoning and inhibiting pathogenic microorganisms during processing and storage. Table 1 presents the OD values of the LAB at various NaCl concentrations. As the NaCl concentration increased, the OD value dropped, showing that the strain’s development had slowed. After the NaCl content was 3%, the ZF8 OD value was 0.91 ± 0.15, and there was a significant difference between the strains (*p* < 0.05). The OD values of ZF22 and TR1-1-3 were significantly higher than those of other strains (*p* < 0.05) when the NaCl concentration reached 6%, the OD value was 0.14 ± 0.01, and the growth ability was the best. When the NaCl concentration reached 12%, the OD values of the five strains decreased, indicating that the strains had hardly grown. This is consistent with Drosinos et al. (27), who discovered that LAB could not grow or function well in high salt concentrations.

**TABLE 1:** Results of lactic acid bacteria tolerance to different salt concentrations.

Strains	0%	3%	6%	7.5%	9%	12%
	OD_600_ values					
ZF6	1.21 ± 0.02Ca	0.19 ± 0.00Cb	0.12 ± 0.01BCc	0.12 ± 0.01Ac	0.12 ± 0.00Ac	0.09 ± 0.01Ad
ZF8	1.54 ± 0.01Aa	0.91 ± 0.15Ab	0.11 ± 0.00Cc	0.13 ± 0.03Ac	0.12 ± 0.01Ac	0.09 ± 0.01Ac
ZF13	1.26 ± 0.02Ba	0.48 ± 0.17Bb	0.11 ± 0.01Cc	0.11 ± 0.00Ac	0.10 ± 0.03Ac	0.09 ± 0.01Ac
ZF22	1.50 ± 0.03Aa	0.86 ± 0.08Ab	0.14 ± 0.01Ac	0.14 ± 0.00Ac	0.12 ± 0.01Ac	0.11 ± 0.00Ac
TR1-1–3	1.52 ± 0.03Aa	0.78 ± 0.03Ab	0.14 ± 0.01ABc	0.12 ± 0.00Acd	0.12 ± 0.01Acd	0.10 ± 0.01Ad

The results are expressed as the mean ± SD (*n* = 3). Uppercase indicates the same condition, and the difference between different samples is statistically significant (*p* < 0.05). Lowercase letters indicate significant differences under different conditions in the same sample (*p* < 0.05).

### 3.2. Lactic acid bacteria growth at different pH values

According to Table 2, the 5 strains could grow at pH values of 3.5, 4.5, 5.5, and 6.5. The OD value increased as the pH raised, showing that the strains had varying degrees of tolerance to different pH levels and that there was a statistically significant difference between pH levels (*p* < 0.05). The OD value of 5 strains was less than 0.5 when pH was 3.5, indicating that strain growth was inhibited. Laslo et al. (21) reported that isolated LAB could not grow at pH 3, which is consistent with our findings. At pH 4.5, 5.5, and 6.5, ZF8, ZF22, and TR1-1-3 all had OD values greater than 1, demonstrating that the bacterial density was greater than that of other strains (*p* < 0.05).

**TABLE 2:** Determination of the tolerance of lactic acid bacteria to different pH values.

Strains	pH3.5	pH4.5	pH5.5	pH6.5
	OD_600_ values			
ZF6	0.23 ± 0.01Dd	0.71 ± 0.07Cc	1.16 ± 0.01Cb	1.23 ± 0.01Ca
ZF8	0.32 ± 0.01Ad	1.11 ± 0.05Ac	1.48 ± 0.01Ab	1.53 ± 0.12Aa
ZF13	0.24 ± 0.03Dd	0.73 ± 0.04Cc	1.19 ± 0.02Cb	1.35 ± 0.01Ba
ZF22	0.44 ± 0.11Ad	1.05 ± 0.07Ac	1.39 ± 0.05Bb	1.53 ± 0.00Aa
TR1-1–3	0.31 ± 0.01BCd	1.08 ± 0.06Ac	1.49 ± 0.01Ab	1.60 ± 0.01Aa

The results are expressed as the mean ± SD (*n* = 3). Uppercase indicates the same condition, and the difference between different samples is statistically significant (*p* < 0.05). Lowercase letters indicate significant differences under different conditions in the same sample (*p* < 0.05).

### 3.3. Lactic acid bacteria growth at different nitrite concentrations

According to Noel et al. (28), adding nitrite to fermented sausages prevented *Salmonella* and *Clostridium’s* growth, contributed to the development of color, inhibited the acidification of fatty acids, and gave the sausages their distinctive cured flavor. The maximum amount of nitrite allowed in fermented meat products is 150 mg/kg, according to GB 2760-2014 Food Safety National Standards for the Usage of Food Additives. LAB is used as a starter in fermented sausages, so they must tolerate nitrite. The nitrite tolerance of LAB is shown in Table 3. The five LAB strains’ growth ability reduced as the nitrite concentration increased, and there were significant variations between concentrations (*p* < 0.05). This is comparable to Verluyten et al. (23), who discovered that even at 10 mg/kg concentrations of sodium nitrite, the development of some LAB is inhibited. When the nitrite concentration was increased to 150 mg/kg, the OD of the five strains fell, and the OD of ZF22 was 1.56 ± 0.03, with the highest growth potential. When the nitrite concentration was 300 mg/kg, there was a significant difference in growth ability between the five strains (*p* < 0.05), and three strains had OD >1, namely ZF22, TR1-1-3, and ZF8, with ZF22 (OD value of 1.48) having the best growth ability.

**TABLE 3:** Determination results of lactic acid bacteria tolerance to different NaNO_2_ concentrations.

Strains	0 mg/kg	50 mg/kg	100 mg/kg	150 mg/kg	200 mg/kg	300 mg/kg
	OD_600_ values					
ZF6	1.29 ± 0.01Ca	1.3 ± 0.04Ca	1.19 ± 0.02Bb	1.06 ± 0.04Cc	1.02 ± 0.02Dc	0.88 ± 0.04Dd
ZF8	1.5 ± 0.03Aa	1.52 ± 0.02Ba	1.52 ± 0.03Aa	1.47 ± 0.02Ba	1.44 ± 0.04Ba	1.11 ± 0.12Cb
ZF13	1.33 ± 0.03Ca	1.33 ± 0.02Ca	1.17 ± 0.01Bb	1.1 ± 0.06Cc	1.07 ± 0.04Cc	0.84 ± 0.03Dd
ZF22	1.43 ± 0.01Be	1.62 ± 0.02Aa	1.53 ± 0.02Ac	1.56 ± 0.03Abc	1.57 ± 0.01Ab	1.48 ± 0.03Ad
TR1-1–3	1.43 ± 0.04Bb	1.53 ± 0.03Ba	1.53 ± 0.02Aa	1.52 ± 0.01ABa	1.47 ± 0.01Bab	1.25 ± 0.09Bc

The results are expressed as the mean ± SD (*n* = 3). Uppercase indicates the same condition, and the difference between different samples is statistically significant (*p* < 0.05). Lowercase letters indicate significant differences under different conditions in the same sample (*p* < 0.05).

### 3.4. Analysis of bacteriostatic characteristics

Table 4 shows that five lactic acid bacterial strains inhibit the four categories of pathogenic bacteria to various degrees. The inhibitory capacities of the five strains on the four types of pathogenic bacteria are as follows: The inhibition circle of *Escherichia coli* was 13.46–17.19 mm, that of *Staphylococcus aureus* was 13.65–17.53 mm, that of *Bacillus subtilis* was 13.62–16.63 mm and that of *Salmonella* was 14.68–17.62 mm. ZF22 inhibited *Escherichia coli* and *Staphylococcus aureus* the most, with antibacterial diameters of 17.19 and 17.53 mm, respectively, that were substantially different from other strains (*p* < 0.05). TR1-1-3 inhibited *Salmonella* and *Bacillus subtilis* the most, with inhibitory diameters of 17.62 and 16.63 mm, respectively, which were substantially different from other strains (*p* < 0.05). Finally, ZF22 and TR1-1-3 show a more substantial antibacterial effect. Our bacteriostatic findings agree with Bungenstock et al. (29) study, which discovered that LAB have antibacterial effects on key foodborne pathogens.

**TABLE 4:** Bacteriostatic ability of lactic acid bacteria.

Strains	*Escherichia coli*	*Staphylococcus aureus*	*Bacillus subtilis*	*Salmonella*
	Diameter of the inhibition circle (mm)			
ZF6	14.62 ± 0.31Cc	13.65 ± 0.25Bd	15.44 ± 0.24Bb	16.89 ± 0.38Aa
ZF8	15.39 ± 0.74BCa	14.33 ± 0.22Bb	15.00 ± 0.46Bab	15.26 ± 0.41Bab
ZF13	13.46 ± 0.75Dc	14.15 ± 0.29Bab	13.62 ± 0.40Cab	14.68 ± 0.67Ba
ZF22	17.19 ± 0.45Ab	17.53 ± 0.74Aa	15.71 ± 0.51Bc	16.99 ± 0.93Aab
TR1-1–3	15.62 ± 0.13Bc	14.20 ± 0.22Bd	16.63 ± 0.38Ab	17.62 ± 0.54Aa

The results are expressed as the mean ± SD (*n* = 3). Uppercase indicates the same condition, and the difference between different samples is statistically significant (*p* < 0.05). Lowercase letters indicate significant differences under different conditions in the same sample (*p* < 0.05).

TR1-1-3 and ZF22, two *Lactobacillus helveticus* strains, showed improved salt, acid, and nitrate tolerance. Additionally, *Lactobacillus helveticus* ZF22 demonstrated the most excellent inhibition of *S. aureus* and *E. coli*, whereas *Lactobacillus helveticus* TR1-1-3 revealed the most significant inhibition of *Bacillus subtilis* and *Salmonella.* Further investigation was carried out using these two strains.

### 3.5. Bacteria and count and pH changes

As shown in the Table 5, Population (lg CFU/g) evolution of LAB in sausages non-inoculated and inoculated with both LAB during fermentation and storage from 0 to 30 days. The trend of changes in the LAB population in ZR, TR1-1-3 and ZF22 groups is the same, all of them have a rapid increase of the LAB population at the beginning of processing, and then gradually decrease. On day 0, the TR-1-3 and ZF22 groups had 10^5^ CFU/g of the LAB population, significantly higher than the ZR group (*p* < 0.05). At the end of fermentation (3 days), the LAB population reached a maximum of 10^8^ CFU/g, with the TR1-1-3 and ZF22 groups significantly higher than the control group (*p* < 0.05). Subsequently, the LAB population decreased due to lower processing temperatures and moisture content. At the end of the ripening period (10 days), the LAB population in the 3 groups of fermented sausages was 10^7^ CFU/g. During storage, the LAB population in the 3 groups of fermented sausages decreased slowly and after 30 days of storage, the LAB population in the 3 groups of fermented sausages tended to be the same. These findings confirm those of Essid et al. (30), who reported that LAB dominates and remains stable or declines slightly during the first 2 weeks of ripening.

**TABLE 5:** Population (lg CFU/g) evolution of lactic acid bacteria (LAB) in sausages non-inoculated and inoculated with both lactic acid bacteria during fermentation and storage from 0 to 30 days.

Group	0 day	3 days	7 days	10 days	15 days	30 days
ZR	4.13 ± 0.04Bd	8.06 ± 0.01Ca	7.64 ± 0.06Ab	7.36 ± 0.04Cc	7.55 ± 0.04Ab	7.59 ± 0.07Ab
TR1-1-3	5.7 ± 0.02Ae	8.92 ± 0.03Aa	7.25 ± 0.05Bd	7.69 ± 0.05Ab	7.55 ± 0.03Ac	7.53 ± 0.08Ac
ZF22	5.7 ± 0.08Ad	8.76 ± 0.01Ba	7.22 ± 0.04Cc	7.58 ± 0.02Bb	7.59 ± 0.04AB	7.5 ± 0.4Abc

The results are expressed as the mean ± SD (*n* = 3). Uppercase indicates the same condition, and the difference between different samples is statistically significant (*p* < 0.05). Lowercase letters indicate significant differences under different conditions in the same sample (*p* < 0.05).

**FIGURE 1:**
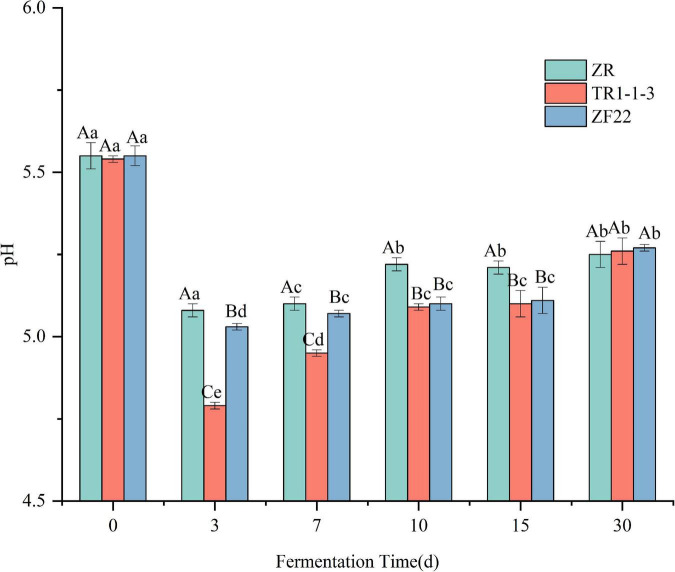
Evolution of pH value in sausages non-inoculated and inoculated with both lactic acid bacteria during fermentation and storage from 0 to 30 days. Error bars refer to the standard deviations obtained from triplicate sample analysis. Uppercase indicates the same condition, and the difference between different samples is statistically significant (*p* < 0.05). Lowercase letters indicate significant differences under different conditions in the same sample (*p* < 0.05).

Figure 1 shows that the changes in pH of the three groups of sausages during the processing and storage of fermented sausages are basically the same, with a trend of decreasing, then increasing and then leveling off. The pH of all groups dropped rapidly from around 5.5 to around 5.0 at the end of fermentation (3 days), significantly lower than the pH at 0 d of fermented sausage (*p* < 0.05), with the TR1-1-3 and ZF22 groups reaching 4.79 and 5.03 respectively, significantly lower than the ZR group (pH = 5.08). This is because inoculation with LAB facilitates the metabolism of the carbohydrates in the fermented sausage to organic acids, such as lactic acid (26), resulting in a rapid reduction in pH. During the subsequent drying and ripening periods (7 and 10 days), the pH tends to increase. This is due to the protein hydrolysis activity leading to the formation of amino acids and peptides that are eventually metabolized by LAB, which may lead to the accumulation of non-protein nitrogen-based compounds and ammonia (31), and these metabolites lead to an increase in pH of the fermented sausages. The pH of fermented sausages increased more slowly during storage (15–30 days), reaching 5.27 (ZR), 5.26 (TR1-1-3), and 5.27 (ZF22) at 30 days.

### 3.6. Analysis of volatile flavor percentage of fermented sausages

**FIGURE 2:**
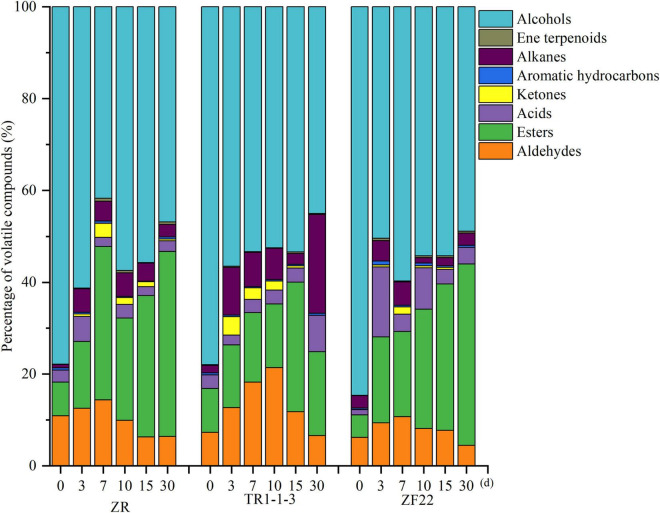
Percentage of volatile substances in fermented sausages. A total of 0, 3, 7, 15, and 30 indicate the processing time of fermented sausages.

Figure 2 depicts the types and amounts of volatile compounds found in fermented sausages by GC–MS analysis. At the processing and storage stage of fermented sausages, the ZR and inoculation groups included primarily alcohols, which may be attributed to the introduction of liquor during fermentation. In addition to alcohols, esters made up a significant portion of the ZR group, followed by aldehydes and alkanes. Esters grew from 22.26% (10 days) to 40.30% (30 days), alcohol content reduced from 57.39% (10 days) to 46.85% (30 days), and acid content decreased from 2.99% (10 days) to 2.35% (30 days). It suggests that alcohols and acids can react chemically to form esters. It has been observed that alcohols and acids are necessary for ester formation (32).

After inoculation, the volatile component concentration of fermented sausages altered dramatically. Except for alcohols, lipid compounds comprised 9.51–28.21% of total volatile chemicals, while aldehydes comprised 6.57–21.40% of total volatile substances in the TR1-1-3 group. The TR1-1-3 group had higher levels of alkanes and ketones than the ZR group. The proportion of alkanes reached a high of 21.54% after 30 days, whereas the proportion of ketones reached a high of 12.96% after 3 days. Compared to the ZR group, the ZF22 group included more acids and ketones; the proportion of acids reached a high of 15.24% after 3 days, while the proportion of ketones reached a maximum of 23.39% at 10 days. Inoculation with LAB impacts the synthesis of volatile chemicals (14).

### 3.7. Volatile flavor composition of fermented sausages with GC–MS

**FIGURE 3:**
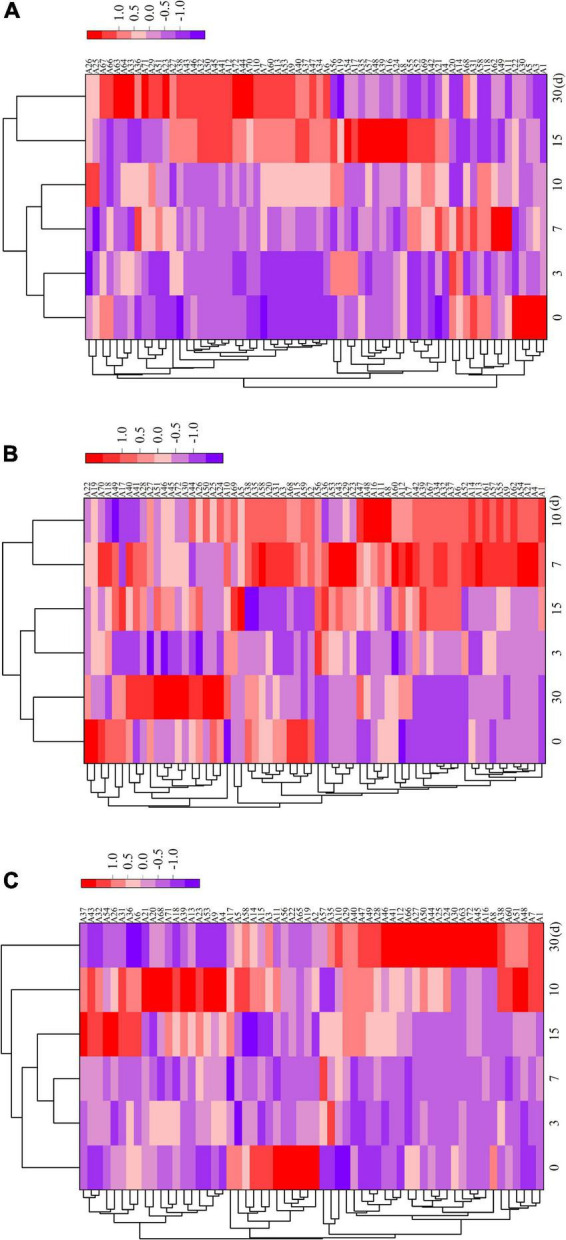
Heat map of ZR fermented sausages from 0 to 30 days and hierarchical clustering analysis of volatile compounds. Rows and columns represent volatiles and time, respectively. **(A)** Heat map of volatile compounds in ZR, **(B)** heat map of volatile compounds in TR1-1-3 and **(C)** heat map of volatile compounds in ZF22. Blue to red shades correspond to volatiles from low to high abundance. A1–A72 are the types of volatiles.

Supplementary Table 1 displays the relative amounts of volatile flavor components in the fermented sausages as determined by GC–MS analysis. After storage (30 days), 72 volatile chemicals, including 11 aldehydes, 11 alcohols, 11 acids, 18 esters, 4 ketones, 3 aromatic hydrocarbons, 10 alkanes, and 4 terpenes, were discovered in all sausage samples. In total, 66, 66, and 60 taste compounds were discovered and quantified in ZR, TR1-1-3, and ZF22 samples, demonstrating that *Lactobacillus helveticus* inoculation impacted the synthesis of volatile compounds. During sausages ripening, these compounds may be produced by enzymatic events (i.e., glycolysis, proteolysis, and oxidative deamination) or chemical reactions (i.e., Maillard reaction, Strecker degradation, and lipid autooxidation) (33). As illustrated in Figure 3, cluster heat map analyses were carried out to analyze the variations in volatile meat content throughout fermentation. Compared to ZF22, volatile chemicals ZR and TR1-1-3 may be classified into three groups using a heat map. The volatile compound trend in TR1-1-3 and ZF22 fermented sausages substantially slowed after 15 days of storage, suggesting that *Lactobacillus helveticus* can accelerate sausages maturation and enhance volatile compound concentration.

In ZR, TR1-1-3, and ZF22, 11 aldehydes were detected, as indicated in Figure 3 and Supplementary Table 1. As the ripening period extended, the types of aldehydes increased. These aldehydes are precursors to low molecular weight volatile compounds that play an essential role in fermented sausage’s flavor development (8, 34). The quantity of heptanaldehyde in TR1-1-3 (range from 0.1379 to 1.07997 μg/kg) is greater than that in ZR, and heptanal has a fruit and herb odor, corresponding with the findings of Yang et al. (5). caproaldehyde and decanal, which give fermented meat its odor of green grass and fat, have also been found; they are derived from the oxidation of linoleic acid and linolenic acid and play a crucial part in the taste development of fermented sausages (35, 36). The relatively high levels of two amino acid products (benzaldehyde and phenylacetaldehyde) in fermented sausages inoculated with LAB appear to be associated with LAB inoculation. This result is consistent with the findings of Kargozari et al. (37). Other high molecular weight aldehydes found in inoculated sausages, such as *trans-*2-decanal, *trans-*2-octenal, and caprylaldehyde, are essential for flavor development because they act as precursors of low molecular weight volatile compounds.

Chinese dry sausage’s distinctive flavor is largely derived from alcohols, especially aliphatic alcohols, which are decreased by aldehydes produced by the oxidation of fat and amino acids (38). The β oxidation and hydrolysis of free fatty acids generate equivalent β-ketoic acids, which are then converted into secondary alcohols (34). Figure 3 and Supplementary Table 1 indicate that fermented sausages contain eleven alcohol flavor compounds. The esterification of alcohols and acids to create esters may cause the initial spike and drop in alcohol concentration. The greatest ethanol concentration among the three sample groups may have resulted from the inclusion of Baijiu during sausages manufacturing. Alcohol is essential to the production of sausages taste (39). Compared to ZR, 1-octen-3-ol appeared in TR1-1-3 and ZF22 at 0.0549 and 0.0707 μg/kg respectively after 15 days storage in fermented sausages. This may result from glucose fermentation and lipid oxidation involving microorganisms (40). One of the key elements of the distinctive flavor of fermented sausages is the fruity-tasting isoamyl alcohol. Alcohols do not add anything to the flavor of the fermented sausages and have a high olfactory threshold.

Organic acids such as acetic acid and butyric acid are volatile chemicals generated by microbial carbohydrate fermentation (41). Due to their pungent odor and low threshold, short-chain fatty acids (acetic acid, butyric acid, caproic acid, octanoic acid, capric acid, and benzoic acid) significantly influence taste development. Figure 3 and Supplementary Table 1 demonstrate that fermented sausages contain eleven distinct acidic flavor compounds. The sour flavor of fermented sausages is linked to acetic acid. Acetic acid is more prevalent in ZR than TR1-1-3 and ZF22 throughout the process. LAB may produce acetic acid by metabolizing pyruvate (42). Sausages fermented with LAB during production contained more butyric acid than sausages that had been naturally fermented. The increased concentration of Octanoic acid in ZR compared to TR1-1-3 and ZF22, which may reach 0.1004 μg/kg after 15 days, suggests that the flavor of naturally fermented sausages may be more stimulating than that of sausages fermented with LAB. Due to the addition of LAB to the fermented sausage, the sorbic acid content is increased. In comparison to TR1-1-3 and ZR, ZF22 had the highest sorbic acid concentration at 30 days, reaching 0.2747 μg/kg. Additionally, the fermented sausages were found to contain oleic acid. ZR had a lower amount than TR1-1-3 and ZF22, and oleic acid can relax blood vessels.

Esters are partially formed from the esterification of different alcohols and acids; most esters have a fruit or floral aroma and impart a distinctive flavor to meat products (15, 43). LAB can also impact the rate of fat oxidation in fermented sausages. Figure 3 and Supplementary Table 1 demonstrate that fermented sausages contain 18 ester flavoring compounds with varying concentrations. In this study, the most prevalent esters in fermented sausages are the ethyl esters, ethyl decanoate, ethyl 2-methyl butyrate, ethyl laurate, ethyl 3-hydroxybutyrate, ethyl acetate, ethyl palmitate, ethyl isobutyrate, ethyl isovalerate, and ethyl valerate. Ethyl ester contributes significantly to the distinctive scent of sausages by providing a fruity taste and hiding acidity (44). At 30 days, TR1-1-3 and ZF22 had more significant amounts of ethyl isobutyrate, ethyl acetate, and ethyl valerate. These chemicals give fermented sausages a fruity, sweet, and flowery taste. However, we discovered that TR1-1-3 and ZF22 had lower levels of ethyl acetate and ethyl decanoate than ZR. As reported by Procopio et al. (45), these data show that ester synthesis is very strain-dependent.

Ketones are generally regarded as byproducts of lipid oxidation, alkane breakdown, and secondary alcohol dehydrogenation (46). As indicated in Figure 3 and Supplementary Table 1, four ketone molecules, including 6-dodecanone, 2-non-anone, 2-heptanone, and 6-methyl-2-heptanone, were found in the sample. Ordóñez et al. (47) revealed that methyl ketones (such as 2-non-anone and 2-heptanone) could develop during lipid oxidation, imparting rose and tea aromas to beef products. The concentration of 2-non-anone (0.0265 to 0.0863 μg/kg) in TR1-1-3 is greater than that of ZR and ZF22. Ten alkanes and three aromatic hydrocarbons have also been discovered in fermented sausages. Hydrocarbons are primarily produced by lipid autooxidation (32). The novel hydrocarbons (2,3-epoxyheptane) were exclusively detected in TR1-1-3 and ZF22 sausages, which may be the result of Lactobacillus helveticus’ engagement in lipid autoxidation. However, the concentration of hydrocarbons is relatively low, and the threshold is quite high, so the effect on the flavor production of fermented sausages is minimal. Chen et al. (36) reported that the 2-pinene, α-Caryophyllene, D limonene and γ-terpene may be derived from spices. However, four terpenes [2-pinene, α-Caryophyllene, S-(-)-limonene and δ-Elemene] were found at low levels in fermented sausages.

### 3.8. Identification of samples based on principal component analysis

**FIGURE 4:**
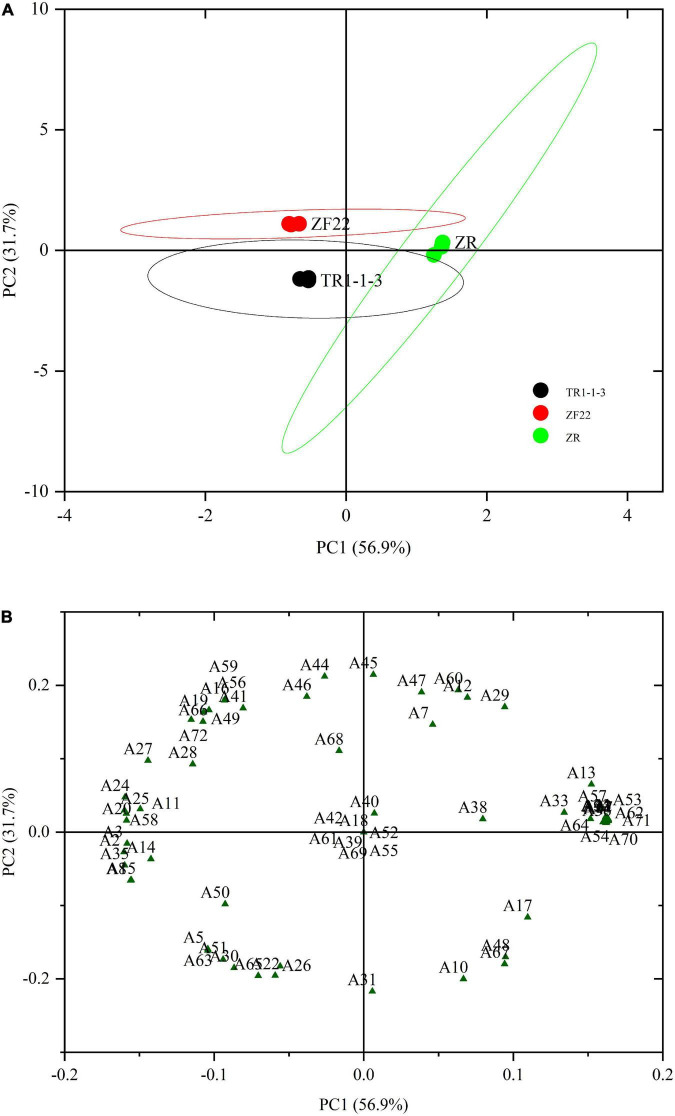
Principal component analysis (PCA) plots of volatile compounds in fermented sausages stored for 30 days. **(A)** PCA score plot depicting the distribution of samples for the first two principal components (PC1 and PC2). **(B)** PCA loading plot depicting the distribution of the 72 volatile compounds for the first two main components (PC1 and PC2). A1–A72 are the types of volatiles.

Principal component analysis is an unsupervised grouping method that highlights changes in volatile compounds by using signal intensity (48). The PCA approach can be successful if the variance proportion is equal to or more than 70%. When fermented sausages were aged for 30 days, the abundance of 72 taste components was analyzed using PCA to distinguish between ZR, TR1-1-3, and ZF22 (Figure 4). A total of 88.6% contribution was made by the first two primary components (56.9% for PC1 and 31.7% for PC2). The findings of the score plot (Figure 4A) demonstrate that ZR, TR1-1-3, and ZF22 are distinguishable in a substantially autonomous space in the distribution plot. ZR was recovered from samples of TR1-1-3 and ZF22 on PC1, whereas TR1-1-3 was isolated from samples of ZF22 on PC2, indicating that PC1 and PC2 are the most distinguishing variables in fermented sausages varieties.

According to the loading graph analysis shown in Figure 4B, ZR was found to locate in the positive region of PC1 and the neutral region of PC2, where decanal (6), (2Z, 5Z)-2, 5-Pentadiene-1-ol (13), 1-octyne-3-ol (17), isoamyl alcohol (19), hexadecanoic acid (23), octanoic acid (32), eicosapentaenoic acid (33), ethyl caprate (34), 1-methyl undecyl acrylate (36), allyl 2-ethyl butyrate (38), ethyl laurate (40), butyl sorbate (43), amyl cyclopropane (64), α-caryophyllene (70), δ-elemene (71) are mainly distributed in this fermented sausages. Samples of TR1-1-3 were found to locate in the PC1-negative and PC2-negative regions, and their characteristic flavor substances were valeraldehyde (5), benzaldehyde (8), 1-pentene-3-ol (14), 1-cyclobutene-1-methanol (15), ethanol (22), butyric acid (26), pterin-6-carboxylic acid (30), methyl sorbate (35), ethyl isovalerate (50), ethyl valerate (51), 1,1-diethoxypropane (63), trichloromethane (65). ZF22 is in the negative zone of PC1 and the positive zone of PC2. ZF22 is in the negative zone of PC1 and the positive zone of PC2. *Trans-*2-octenal (11), isoamyl alcohol (19), hexanol (20), sorbic acid (24), oleinic acid (25), l-cysteine (27), myristic acid (28), ethyl 3-hydroxybutyrate (41), isopropyl trimethylsilyl disilicate (44), isoamyl acetate (46), ethyl iso-butyrate (49), 1,1-diethoxy ethane (56), 1,2-epoxy-cycloheptane (58), 2,3-epoxyheptane (59), o-isopropyl toluene (68), δ-elemene (72) is a variable associated with ZF22. Finally, PCA can identify different types of fermented sausages. However, PCA can also determine the necessary classification basis using the distribution of load factors, enabling a rapid technique of fermented sausages differentiation.

## 4. Conclusion

In this study, an analysis of Lactobacillus tolerance revealed that ZF22 and TR1-1-3 demonstrated more excellent salt, acid, and nitrite tolerance. Analysis of bacterial inhibition revealed that ZF22 exhibited the highest inhibition of *E. coli* and *S. aureus*, whereas TR1-1-3 showed significant inhibition of *B. subtilis* and *Salmonella*. Analysis of volatile flavor compounds showed that 72 volatile chemicals were found in all sausage samples, including 11 aldehydes, 11 alcohols, 11 acids, 18 esters, 4 ketones, 3 aromatic hydrocarbons, 10 alkanes and 4 terpenes, with an increased proportion of acids, alkanes, and ketones in TR1-1-3 and ZF22, and no significant increase was found in the natural group. Esters such as ethyl isobutyrate, ethyl acetate, and ethyl valerate became common in TR1-1-3 and ZF22 fermented sausages, according to cluster analysis. In addition, the outcomes of the PCA indicated that the various groups of fermented sausages were highly distinctive, giving a quick approach to differentiate fermented sausages. Therefore, based on the current investigation findings, we propose that *Lactobacillus helveticus* TR1-1-3 and ZF22 can be employed as fermenting agents for fermented sausages to improve the flavor and safety of fermented sausages.

## Data availability statement

The original contributions presented in this study are included in the article/Supplementary material, further inquiries can be directed to the corresponding author.

## Author contributions

JT: writing – review and editing, validation, acquired funding, and project administration. XY: data curation and writing – original draft. KZ: formal analysis and investigation. YZ: methodology. FC: conceptualization. YJ: supervision. All authors contributed to the article and approved the submitted version.
