# Supplementary material for: Influence of Lactobacillus helveticus ZF22 and TR1-1-3 strains on the aromatic flavor of fermented sausages
Source: Front Nutr. 2023 Jan 9;9:1058109. doi: 10.3389/fnut.2022.1058109 (PMC9868738; doi:10.3389/fnut.2022.1058109)
Supplement: Supplementary file 1 [file Table_1.DOCX]

Supplementary Material

## Supplementary TABLE 1 Volatile flavour compounds identified and quantified (µg/kg) in fermented sausages by GC–MS during storage time

| flavour compounds | group | 0d | 3d | 7d | 10d | 15d | 30d |
| --- | --- | --- | --- | --- | --- | --- | --- |
| **Aldehydes** |  |  |  |  |  |  |  |
| Isovaleraldehyde (A1) | ZR | ND | ND | ND | 0.0163±0.0025Cb | 0.0234±0.0032Aa | ND |
|  | TR1-1-3 | ND | 0.0097±0.0015Ac | 0.0358±0.0061Aa | 0.0403±0.003Aa | 0.0200±0.0006Bb | ND |
|  | ZF22 | ND | ND | 0.0090±0.0015Bc | 0.0316±0.0016Bb | ND | 0.0351±0.0011Aa |
| Caproaldehyde (A2） | ZR | 0.3231±0.0287ABa | 0.0963±0.0441Ab | 0.0950±0.0165Bb | 0.0977±0.0049Bb | ND | ND |
|  | TR1-1-3 | 0.4212±0.1192Ba | 0.1100±0.0116Ab | 0.4463±0.0428Aa | 0.4653±0.0401Aa | ND | 0.1077±0.0153Ab |
|  | ZF22 | 0.3052±0.0904ABa | 0.1152±0.0187Ab | 0.0767±0.0394Bb | 0.1349±0.0055Bb | ND | 0.1011±0.0184Ab |
| Heptaldehyde (A3) | ZR | 0.3443±0.0424Ba | 0.1396±0.0754Abc | 0.2053±0.0781Bb | 0.1854±0.0138Bb | 0.1598±0.0575Abc | 0.0651±0.0012Bc |
|  | TR1-1-3 | 0.6090±0.1632Ab | 0.1465±0.0291Ac | 1.0797±0.1226Aa | 0.9441±0.0671Aa | 0.1379±0.0099Ac | 0.2599±0.0500Ac |
|  | ZF22 | 0.3060±0.1120Ba | 0.1268±0.0062Abc | 0.1523±0.0427Bbc | 0.2194±0.0148Bab | 0.1030±0.0417Ac | 0.2595±0.037Aa |
| Trans-2-decenal (A4) | ZR | ND | ND | 0.0099±0.0023Ba | 0.0083±0.0006Ca | 0.0090±0.0006Aa | 0.0083±0.0001Aa |
|  | TR1-1-3 | ND | 0.0040±0.0003Ac | 0.0328±0.0103Aa | 0.0188±0.0026Ab | 0.0049±0.0015Bc | ND |
|  | ZF22 | ND | 0.0062±0.0021Ab | 0.0039±0.0002Bc | 0.0150±0.0011Ba | 0.0057±0.0003Bb | ND |
| Valeraldehyde (A5) | ZR | 0.3443±0.0045ABa | 0.0161±0.0014Ae | 0.0414±0.0023Bb | 0.0339±0.0023Bc | 0.0369±0.0058Abc | 0.0236±0.0025Cd |
|  | TR1-1-3 | 0.0621±0.0181Aa | 0.0181±0.0027Aa | 0.1212±0.0119Aa | 0.1262±0.0100Aa | 0.4056±0.6509Aa | 0.0559±0.0057Aa |
|  | ZF22 | 0.0346±0.0071BCb | ND | 0.0179±0.0074Cc | 0.0476±0.003Ba | 0.0135±0.006Ac | 0.0322±0.0033Bb |
| Decanal (A6) | ZR | ND | 0.1230±0.0334Ad | 0.2054±0.0346Bc | 0.4473±0.0115Ab | 0.5630±0.0285Aa | 0.5937±0.0455Aa |
|  | TR1-1-3 | ND | 0.1433±0.0096Ab | 0.4239±0.0577Aa | 0.4169±0.0418Aa | 0.4278±0.0270Ba | ND |
|  | ZF22 | 0.3560±0.0559Ac | 0.1709±0.0279Ad | 0.2004±0.0157Bd | 0.4410±0.0074Ab | 0.5434±0.0123Aa | ND |
| Phenylacetaldehyde (A7) | ZR | ND | 0.0128±0.0054Aa | 0.0349±0.0093Bc | 0.0374±0.0015Cc | 0.0480±0.0014Ab | 0.0611±0.0036Aa |
|  | TR1-1-3 | ND | 0.0162±0.0015Ad | 0.1242±0.0337Aa | 0.0659±0.0101Bb | 0.0370±0.0041Bcd | 0.0543±0.0047Abc |
|  | ZF22 | ND | 0.0191±0.0054Ac | 0.0207±0.0039Bbc | 0.0665±0.003Ba | 0.0265±0.0025Cb | 0.0612±0.0039Aa |
| Benzaldehyde (A8) | ZR | 0.1055±0.0260Bb | 0.0859±0.0141Ab | ND | ND | 0.2498±0.0349Aa | ND |
|  | TR1-1-3 | 0.1834±0.0182Abc | 0.0142±0.0005Ad | 0.3205±0.2915Ab | 0.7906±0.0575Aa | ND | 0.2433±0.0181Ab |
|  | ZF22 | 0.1164±0.0264Bb | ND | 0.0128±0.0024Bc | ND | ND | 0.1841±0.0182Ba |
| Caprylaldehyde (A9) | ZR | 0.0630±0.0090Be | 0.049±0.0113Ce | 0.1315±0.0075Bd | 0.1740±0.0090Cc | 0.2318±0.0188Ab | 0.2654±0.0165Aa |
|  | TR1-1-3 | 0.092±0.0118Ade | 0.1166±0.0115Bd | 0.3160±0.0362Aa | 0.2793±0.0173Ab | 0.1554±0.0094Bc | 0.0655±0.0067Ce |
|  | ZF22 | 0.0793±0.0203ABc | 0.1343±0.0023Bb | 0.1213±0.0359Bb | 0.2124±0.0064Ba | 0.1215±0.0126Cb | 0.0720±0.0022BCc |
| 3-Hydroxybutyraldehyde (A10) | ZR | 0.0049±0.0009Ad | 0.0200±0.0029Bc | 0.0253±0.0065Bc | 0.0236±0.0023Bc | 0.0634±0.0041Ab | 0.0820±0.0089Ba |
|  | TR1-1-3 | ND | 0.0689±0.0073Ac | 0.0789±0.0022Ab | 0.0641±0.0062Ac | 0.0484±0.0041Bd | 0.0919±0.0014Aa |
|  | ZF22 | ND | 0.0201±0.0013Bbc | 0.0267±0.0097Bc | 0.0154±0.0012Bc | 0.0219±0.0035Cbc | 0.0365±0.0020Ca |
| Trans-2-octenal (A11) | ZR | 0.0141±0.003Cab | 0.0097±0.0038Bbc | 0.0173±0.0009Ba | 0.0113±0.0009Cbc | 0.0124±0.0026Bbc | 0.0088±0.0009Bc |
|  | TR1-1-3 | 0.0258±0.0013ABc | 0.0106±0.0007Bc | 0.0614±0.0211Ab | 0.1045±0.0051Aa | 0.0214±0.0029Ac | 0.0151±0.0019Ac |
|  | ZF22 | 0.0437±0.0230Aa | 0.0175±0.0015Abc | 0.0066±0.0011Bc | 0.0324±0.0008Bb | 0.0186±0.0009Abc | 0.0171±0.0028Abc |
| **Alcohols** |  |  |  |  |  |  |  |
| 2,3 – Butanediol (A12) | ZR | ND | 0.1640±0.0356Cbc | 0.6140±0.3166Bbc | 0.7418±0.2753Cb | 4.1792±0.4770Aa | 4.3568±0.5384Aa |
|  | TR1-1-3 | ND | 0.3249±0.0352Ac | 2.7971±0.5614Aa | 1.7874±0.1434Bb | 2.2261±0.0494Bb | 1.8654±0.0493Bb |
|  | ZF22 | ND | 0.2447±0.0357Be | 0.5391±0.2237Bd | 2.2882±0.0929Ab | 1.6076±0.0806Cc | 4.3115±0.2514Aa |
| (2Z, 5Z) -2, 5-Pentadiene-1-ol (A13) | ZR | 0.0063±0.0026Bd | 0.0053±0.0027BCd | 0.0184±0.0036Bc | 0.0324±0.0006Bb | 0.0374±0.0014Ab | 0.0527±0.0106Aa |
|  | TR1-1-3 | 0.0116±0.0036Ac | 0.006±0.0003ABc | 0.0419±0.0047Aa | 0.0320±0.0080Bb | 0.0137±0.0027Cc | 0.0121±0.0017Bc |
|  | ZF22 | 0.0095±0.0029ABd | 0.0088±0.0021Ad | 0.0095±0.0018Cd | 0.0486±0.0034Aa | 0.0292±0.0023Bb | 0.0208±0.0017Bc |
| 1-pentene-3-ol (A14) | ZR | 0.0042±0.0013Bb | 0.0043±0.0015Ab | 0.0065±0.0012Ba | ND | ND | ND |
|  | TR1-1-3 | 0.0077±0.0014Ab | 0.0041±0.0005Ab | 0.0168±0.0024Aa | 0.0151±0.0006Aa | 0.0076±0.0067Ab | 0.0066±0.0003Ab |
|  | ZF22 | 0.0087±0.0018Aa | 0.0044±0.0008Ab | 0.0031±0.0012Cc | 0.0083±0.0031Aa | ND | 0.0058±0.0029Aab |
| 1-Cyclobutene-1-methanol (A15) | ZR | ND | ND | 0.0232±0.0049Ba | 0.0155±0.0026Bb | ND | ND |
|  | TR1-1-3 | 0.0511±0.0154Aa | 0.0251±0.0036Acd | 0.0440±0.0110Aab | 0.0352±0.0027Abc | 0.0164±0.0047Ad | 0.0209±0.0015Acd |
|  | ZF22 | 0.0380±0.00620ABa | ND | 0.0147±0.0013Bc | 0.0287±0.0015Ab | ND | 0.0159±0.0025Bc |
| Diethylene glycol (A16) | ZR | 0.0346±0.0024Ab | ND | ND | 0.3232±0.0691Bb | 3.6734±0.4021Aa | ND |
|  | TR1-1-3 | 0.0367±0.0043Ac | ND | 1.2101±0.6991Ab | 2.4066±0.4259Aa | 0.6792±0.5596Bbc | ND |
|  | ZF22 | ND | ND | 0.2776±0.0096Bb | ND | 0.0511±0.0126Bc | 1.6288±0.0825Aa |
| 1-Octyne-3-ol (A17) | ZR | 0.0425±0.0071Ab | 0.1023±0.0490Aa | 0.0222±0.0145Ab | 0.0371±0.0035Ab | 0.1160±0.0083Aa | 0.0483±0.0068Ab |
|  | TR1-1-3 | 0.0618±0.0174Aa | ND | ND | ND | 0.0684±0.0041Ba | 0.0435±0.0012ABb |
|  | ZF22 | 0.0473±0.0165Aa | 0.0301±0.0037Bb | ND | 0.0441±0.0015Aab | 0.0483±0.0081Ca | 0.0356±0.003Bab |
| 1-Octene-3-ol (A18) | ZR | 0.0466±0.0084Bb | ND | 0.0594±0.0017Ba | 0.06060±0.009Ba | ND | ND |
|  | TR1-1-3 | 0.0937±0.0019Ab | 0.0601±0.004Bc | 0.1136±0.016Aa | ND | 0.0549±0.008Bc | ND |
|  | ZF22 | 0.0534±0.0214Bc | 0.0843±0.0122Ac | ND | 0.1355±0.0096Aa | 0.0707±0.0096Abc | ND |
| Isoamyl alcohol (A19) | ZR | 0.2253±0.0212Bc | 0.3618±0.0603Aa | 0.2773±0.0255Ab | 0.3650±0.0120Aa | 0.3077±0.0050Bb | 0.0574±0.0088Bc |
|  | TR1-1-3 | 1.6447±0.4568Aa | 0.3341±0.0344Ab | 0.3057±0.0254Ab | 0.2923±0.0296Bb | 0.2801±0.0231Bb | 0.0783±0.0030Bb |
|  | ZF22 | 1.2275±0.1508Aa | 0.3831±0.0342Abc | 0.3169±0.1202Abc | 0.3465±0.0115Abc | 0.4180±0.0344Ab | 0.2342±0.0176Ac |

Supplementary TABLE 1 (*continued*)

| flavour compounds | group | 0d | 3d | 7d | 10d | 15d | 30d |
| --- | --- | --- | --- | --- | --- | --- | --- |
| Hexanol (A20) | ZR | 0.0288±0.0047Bab | 0.0338±0.0107Aa | 0.0249±0.0125Bab | 0.0175±0.0016Bb | 0.0202±0.0010Ab | 0.0162±0.0016Bb |
|  | TR1-1-3 | 0.0459±0.0069Ac | 0.038±0.0047Acd | 0.0765±0.0161Aa | 0.0603±0.0037Ab | 0.0190±0.0008Ae | 0.0280±0.0019Ade |
|  | ZF22 | 0.0302±0.0056Bb | 0.0359±0.0042Ab | 0.0199±0.0043Bc | 0.0573±0.0041Aa | 0.0185±0.0025Ac | 0.0305±0.0033Ab |
| 9- Oxabicyclic [3.3.1] nonane-2, 6-diol (A21) | ZR | ND | 0.0534±0.0248Acd | 0.1172±0.0303Ba | 0.0665±0.0043Bbc | 0.0950±0.0257Aab | 0.0303±0.0041Ade |
|  | TR1-1-3 | 0.1050±0.0223Ac | 0.0513±0.0100Ac | 0.6450±0.2228Aa | 0.4410±0.0817Ab | 0.0432±0.0059Bc | ND |
|  | ZF22 | 0.0679±0.0186Bb | 0.0651±0.0121Ab | 0.0408±0.0033Bc | 0.1893±0.0069Ba | 0.0236±0.0112Bc | ND |
| Ethanol (A22) | ZR | 7.8012±0.6745Ba | 1.7453±0.1959Ad | 1.5019±1.1721Bd | 4.1144±0.4116ACbc | 4.5471±0.5968Ab | 3.2315±0.5799Bc |
|  | TR1-1-3 | 8.7429±0.1238Ba | 2.0921±0.1939Ad | 3.8950±0.3940Abc | 3.0327±0.4114Bcd | 2.3020±1.9532Bd | 4.8744±0.3507Ab |
|  | ZF22 | 15.1575±3.2607Aa | 2.4951±0.0205Ab | 2.2875±0.2055Bb | 4.7544±0.4426Ab | 4.4853±0.3315Ab | 3.1097±0.1978Bb |
| **Acids** |  |  |  |  |  |  |  |
| Hexadecanoic acid (A23) | ZR | ND | ND | 0.0119±0.0049Bb | 0.0058±0.0003Bc | 0.0077±0.0005Cbc | 0.0221±0.0029Aa |
|  | TR1-1-3 | ND | 0.0056±0.0016Bc | 0.0415±0.0057Aa | 0.0049±0.0007Bc | 0.0165±0.0019Bb | ND |
|  | ZF22 | ND | 0.0099±0.0022Ac | 0.0201±0.0022Bb | 0.0305±0.0024Aa | 0.0219±0.0031Ab | ND |
| Sorbic acid (A24) | ZR | 0.0681±0.0063Bb | 0.0513±0.0135Ac | 0.0037±0.0006Bd | 0.0489±0.0021Bc | 0.1570±0.0112Aa | 0.0578±0.0089Cbc |
|  | TR1-1-3 | 0.1164±0.0177Ac | 0.0213±0.0013BCd | 0.0388±0.0059Ad | 0.1448±0.0183Ab | 0.0334±0.0045Cd | 0.2115±0.0167Ba |
|  | ZF22 | 0.0561±0.0055Bc | 0.0107±0.0018Cd | 0.0439±0.0035Ac | 0.1811±0.0075Ab | 0.0496±0.0021Bc | 0.2747±0.0150Aa |
| Oleinic acid (A25) | ZR | 0.019±0.0051Bb | 0.0112±0.0033Ac | 0.0046±0.0021Ad | 0.0301±0.0036Aa | 0.0168±0.0036Ac | 0.0207±0.0037Cb |
|  | TR1-1-3 | 0.0287±0.0076Ab | ND | ND | 0.0142±0.0064Ac | 0.0053±0.0003Bb | 0.0484±0.0026Ba |
|  | ZF22 | ND | ND | 0.0040±0.0008Ac | 0.0189±0.0013Ab | 0.0042±0.0008Bc | 0.0558±0.0040Aa |
| Butyric acid (A26) | ZR | 0.0296±0.0029Bbc | 0.0088±0.0028Bc | 0.0211±0.0001ABbc | 0.0598±0.0175Aa | 0.0421±0.0174Bab | 0.0380±0.0152Bab |
|  | TR1-1-3 | 0.0489±0.0047Abc | 0.0140±0.0021Ad | 0.0313±0.0132ABcd | 0.0461±0.0153Abc | 0.0578±0.0132Bab | 0.0730±0.0074Aa |
|  | ZF22 | 0.0455±0.0048Ab | 0.0126±0.0033ABc | 0.0364±0.0046Abc | 0.0593±0.0115Ab | 0.1111±0.0301Aa | 0.0351±0.0143Bbc |
| L-Cysteine (A27) | ZR | ND | 0.0109±0.0041Aa | 0.0108±0.0054Aa | ND | 0.0142±0.0032Aa | 0.0134±0.0023Ca |
|  | TR1-1-3 | 0.0262±0.0047Ab | 0.0043±0.001Bd | 0.0260±0.0028Ab | 0.0254±0.002Ab | 0.0170±0.0011Ac | 0.0339±0.0037Ba |
|  | ZF22 | 0.0268±0.0033Ab | 0.0061±0.0004Bc | 0.0074±0.0006Bc | 0.0255±0.0010Ab | 0.0100±0.0019Bc | 0.0554±0.0055Aa |
| Myristic acid (A28) | ZR | ND | ND | ND | 0.0123±0.0012Ba | ND | 0.0123±0.0052Ba |
|  | TR1-1-3 | ND | ND | 0.0134±0.0008Ab | ND | 0.0072±0.0014Bc | 0.0164±0.0013ABa |
|  | ZF22 | ND | 0.0045±0.0008Ad | 0.0071±0.0007Bc | 0.0133±0.0004Ab | 0.0113±0.0008Ac | 0.0206±0.0011Aa |
| Dehydroacetic acid (A29) | ZR | 0.0226±0.0033Ab | 0.0172±0.0125Ab | 0.0318±0.0161Bab | 0.0380±0.0014Aab | 0.0215±0.0040Bb | 0.0453±0.0168Aa |
|  | TR1-1-3 | ND | 0.0074±0.0011Acd | 0.0554±0.0131Aa | 0.0139±0.0046Bbc | 0.0210±0.0072Bb | ND |
|  | ZF22 | ND | 0.0183±0.0069Ab | 0.0233±0.0054BCb | 0.0394±0.0038Aa | 0.0347±0.0023Aa | 0.0356±0.0018ABa |
| Pterin-6-carboxylic acid (A30) | ZR | 0.0887±0.0175Aa | 0.0245±0.0075Ab | 0.0030±0.0003Ac | ND | 0.0092±0.0021Bc | 0.0137±0.0005Cbc |
|  | TR1-1-3 | 0.0516±0.0187Bbc | 0.0022±0.0002Cc | 0.1634±0.1831Ab | 0.0118±0.0020Ac | 0.0887±0.0537Abc | 0.7984±0.0088Aa |
|  | ZF22 | 0.0344±0.0074BCb | ND | 0.0077±0.0025Ac | 0.0088±0.0005Ac | 0.0096±0.0014Bc | 0.1075±0.0038Ba |
| Lactic acid (A31) | ZR | 0.0101±0.0014Ba | ND | 0.0102±0.0001Ba | ND | ND | 0.0050±0.0006Bb |
|  | TR1-1-3 | 0.0185±0.0033Ac | ND | 0.0286±0.0031Aa | 0.0241±0.001Ab | ND | 0.0107±0.0003Ad |
|  | ZF22 | ND | 0.0100±0.0005Ab | ND | 0.0126±0.0003Ba | ND | ND |
| Octanoic acid (A32) | ZR | ND | 0.0110±0.0030Ac | 0.0232±0.0032Bb | ND | 0.1004±0.0037Aa | 0.0914±0.0110Aa |
|  | TR1-1-3 | ND | 0.0111±0.0008Ab | 0.0557±0.0198Aa | 0.0428±0.0046Ba | 0.0421±0.0045Ca | ND |
|  | ZF22 | 0.0242±0.0037Ab | ND | ND | 0.0631±0.0019Aa | 0.0637±0.0018Ba | ND |
| Eicosapentaenoic acid (A33) | ZR | 0.0056±0.0018Aab | 0.0060±0.0023Aab | ND | 0.0072±0.0004Aab | ND | 0.0127±0.0092Aa |
|  | TR1-1-3 | 0.0075±0.0003Aa | ND | ND | ND | ND | ND |
|  | ZF22 | ND | ND | ND | ND | 0.0062±0.0030Aa | ND |
| **Esters** |  |  |  |  |  |  |  |
| Ethyl caprate (A34) | ZR | ND | ND | 0.3629±0.1105Bc | 0.7879±0.0752Ab | 0.9785±0.0636Aa | 1.0251±0.1943Aa |
|  | TR1-1-3 | ND | 0.2167±0.0064Ab | 0.7627±0.1767Aa | 0.6276±0.0995Ba | 0.6189±0.0891Ba | ND |
|  | ZF22 | ND | 0.2480±0.0662Ad | 0.3796±0.0514Bc | 0.8764±0.0271ABb | 1.0088±0.0610Aa | ND |
| Methyl sorbate (A35) | ZR | 0.0182±0.0039Ab | ND | 0.0144±0.0024Bb | ND | 0.0479±0.0121Aa | ND |
|  | TR1-1-3 | 0.0185±0.0033Ab | 0.0080±0.001ABc | 0.0274±0.0075Aa | 0.0250±0.0019Aa | ND | 0.0172±0.0016Ab |
|  | ZF22 | ND | 0.0185±0.0104Aa | 0.0060±0.0008Ccd | ND | 0.0092±0.0020Bbc | 0.0147±0.0012Bab |
| 1-Methyl undecyl acrylate (A36) | ZR | ND | 0.0026±0.0007Ccd | 0.0080±0.0045Aa | 0.0041±0.0006Bbc | ND | 0.0072±0.0006Aab |
|  | TR1-1-3 | ND | 0.0062±0.0004Ab | 0.0082±0.0012Aab | ND | 0.0103±0.0039Aa | ND |
|  | ZF22 | 0.0071±0.0007Aab | 0.0073±0.0011Aab | 0.0048±0.0036Ab | 0.0085±0.0016Ab | 0.0105±0.0010Aa | ND |
| Ethyl benzoate (A37) | ZR | ND | ND | 0.0474±0.0128Cd | 0.1014±0.0037Cc | 0.1610±0.0101Ca | 0.1449±0.0121Ab |
|  | TR1-1-3 | ND | 0.0445±0.0052Ab | 0.2571±0.0318Aa | 0.2235±0.0232Ba | 0.2494±0.0226Ba | ND |
|  | ZF22 | ND | 0.0716±0.0149Ad | 0.1255±0.0097Bc | 0.2789±0.0069Ab | 0.4064±0.0098Aa | ND |
| Allyl 2-ethyl butyrate (A38) | ZR | ND | 0.0058±0.0006Abc | 0.0041±0.0006Bc | 0.0053±0.0003Ac | 0.0078±0.0007Aab | 0.0086±0.0028Aa |
|  | TR1-1-3 | 0.0074±0.0039Aa | 0.0027±0.0004Cb | 0.0081±0.0019Aa | 0.0075±0.0008Aa | ND | 0.0064±0.0006Aa |
|  | ZF22 | ND | 0.0036±0.0004Bb | ND | 0.0072±0.0009Aa | ND | 0.0069±0.0010Aa |

Supplementary TABLE 1 (*continued*)

| flavour compounds | group | 0d | 3d | 7d | 10d | 15d | 30d |
| --- | --- | --- | --- | --- | --- | --- | --- |
| Ethyl 2-methyl butyrate (A39) | ZR | ND | ND | 0.0859±0.0330Bb | 0.0707±0.0298Bb | 0.3600±0.0207Aa | ND |
|  | TR1-1-3 | ND | 0.0396±0.0074Ac | 0.2262±0.0225Aab | 0.2099±0.0275Ab | 0.3147±0.1250Aa | ND |
|  | ZF22 | ND | ND | 0.0584±0.0179Bc | 0.2417±0.0165Aa | 0.1310±0.0214Bb | ND |
| Ethyl laurate (A40) | ZR | 0.0855±0.0176Bb | 0.0701±0.0252Ab | 0.1101±0.0291Bb | 0.1771±0.0181Aa | 0.2148±0.0107Aa | 0.2070±0.0391Aa |
|  | TR1-1-3 | 0.1508±0.0239Ab | 0.0790±0.0016Ac | 0.2083±0.0436Aa | 0.0543±0.0086Bc | 0.1254±0.0208Bb | 0.2064±0.0222Aa |
|  | ZF22 | 0.1415±0.0174Ab | 0.0888±0.0226Ac | 0.1063±0.0157Bc | 0.1904±0.0056Aa | 0.1934±0.0124Aa | 0.2062±0.0107Aa |
| Ethyl 3-hydroxybutyrate (A41) | ZR | ND | ND | ND | 0.0059±0.0021BCb | 0.0308±0.0035Aa | 0.0307±0.0029Ba |
|  | TR1-1-3 | ND | ND | 0.0205±0.0078Ab | ND | 0.0231±0.0042Bab | 0.0293±0.0061Ba |
|  | ZF22 | ND | ND | ND | 0.0151±0.0035Ab | 0.0162±0.0024Cb | 0.0493±0.0040Aa |
| 4-Hydroxybutyrate tlactone (A42) | ZR | ND | ND | 0.0052±0.0021Bb | 0.0073±0.0006Aab | 0.0080±0.0019Aa | ND |
|  | TR1-1-3 | ND | 0.0060±0.0011Ab | 0.0102±0.0022Aa | 0.0077±0.0011Ab | 0.0062±0.0001Ab | ND |
|  | ZF22 | ND | ND | 0.0034±0.0011Ba | ND | 0.0040±0.0001Ba | ND |
| Butyl sorbate (A43) | ZR | ND | ND | 0.0386±0.0049Bc | ND | 0.0821±0.0067Ab | 0.0999±0.0173Aa |
|  | TR1-1-3 | ND | 0.0190±0.0011Bc | 0.0615±0.0136Aa | ND | 0.0396±0.0054Bb | ND |
|  | ZF22 | ND | 0.0319±0.0073Ab | 0.0324±0.0024Bb | 0.0713±0.0022Aa | 0.0756±0.0057Aa | ND |
| Isopropyl trimethylsilyl disilicate (A44) | ZR | 0.0039±0.0001Bc | 0.0020±0.0001Bc | 0.0059±0.0018Ac | 0.0050±0.0001Bc | 0.0103±0.0013Ab | 0.0148±0.0022Ba |
|  | TR1-1-3 | 0.0044±0.0002Bb | ND | ND | 0.0074±0.0031Ba | 0.0056±0.0006Bab | 0.0080±0.0014Ca |
|  | ZF22 | 0.0063±0.0010Ac | 0.0034±0.0005Ae | 0.0036±0.0009Ae | 0.0123±0.0013Ab | 0.0054±0.0002Bcd | 0.0241±0.0025Aa |
| Ethyl acetate (A45) | ZR | 0.1205±0.0424Ab | 0.0642±0.0379Ab | 0.1003±0.0837Bb | 0.1359±0.0297Bb | 4.2631±0.3411Aa | 3.9594±0.3255Ba |
|  | TR1-1-3 | 0.2000±0.0778Ad | 0.0894±0.0139Ae | 0.3332±0.0633Ac | 0.3409±0.0834Ac | 0.4656±0.0734Cb | 0.7798±0.0163Ca |
|  | ZF22 | 0.1448±0.0167Ac | 0.0688±0.0014Ac | 0.2516±0.0633Ac | 0.3904±0.0367Ac | 0.8774±0.1075Bb | 6.0208±0.5410Aa |
| Isoamyl acetate (A46) | ZR | 0.0237±0.0007ABc | 0.0067±0.0004Ad | 0.0128±0.0021Bd | 0.0135±0.0016Bd | 0.0694±0.0025Ab | 0.0909±0.0110Ba |
|  | TR1-1-3 | 0.0267±0.0111ABc | 0.0073±0.0005Aa | 0.0358±0.0096Ac | 0.0386±0.0036Ac | 0.0511±0.0064Bb | 0.0777±0.0034Bd |
|  | ZF22 | 0.0160±0.0024Bde | 0.0073±0.0016Af | 0.0250±0.0093ABd | 0.0417±0.0034Ac | 0.0635±0.0062Ab | 0.1137±0.0083Aa |
| Ethyl palmitate (A47) | ZR | 0.0055±0.0016Cd | 0.0129±0.0033Bd | 0.0346±0.0092Bc | 0.0709±0.0091Ab | 0.0842±0.0057Aab | 0.0948±0.0242Aa |
|  | TR1-1-3 | 0.0194±0.0026Abc | 0.0152±0.0019Bc | 0.0571±0.0114Aabc | 0.0743±0.0536Aa | 0.0381±0.0073Babc | 0.0605±0.0085Bab |
|  | ZF22 | 0.0105±0.0037Be | 0.0215±0.002Ad | 0.0342±0.0072Bc | 0.0844±0.0033Ab | 0.0923±0.0078Ab | 0.1031±0.0036Aa |
| Sec-butyl nitrite (A48) | ZR | ND | 0.0016±0.0001Ac | 0.0031±0.0007BCc | ND | 0.0660±0.0061Aa | 0.0172±0.0017Ab |
|  | TR1-1-3 | ND | ND | 0.0195±0.0041Abc | 0.0556±0.0073Aa | 0.0235±0.0001Bb | 0.0168±0.0011Ac |
|  | ZF22 | ND | ND | 0.0060±0.0018Bc | 0.0191±0.0011Ba | 0.0071±0.0005Cc | 0.0115±0.0004Bb |
| Ethyl iso-butyrate (A49) | ZR | 0.0167±0.0017Bab | 0.0128±0.0034Aab | 0.0445±0.0421Aa | 0.0127±0.0045Bab | ND | 0.0157±0.0012Bab |
|  | TR1-1-3 | 0.0290±0.0047Aa | 0.0041±0.0010Bb | 0.0273±0.0038ABa | ND | 0.0286±0.0144Aa | 0.0208±0.0034Ba |
|  | ZF22 | ND | 0.0137±0.0003Ae | 0.0217±0.0062ABd | 0.0498±0.0019Ab | 0.0403±0.0025Ac | 0.0674±0.0075Aa |
| Ethyl isovalerate (A50) | ZR | 0.0158±0.0006ABc | 0.0829±0.0143Ab | ND | 0.0106±0.0016Bc | 0.5695±0.0269Aa | 0.5466±0.0780Aa |
|  | TR1-1-3 | 0.0195±0.0047ABc | ND | ND | 0.3511±0.0384Ac | 0.0058±0.0021Bc | 0.6383±0.0031Aa |
|  | ZF22 | 0.0150±0.0029Bd | 0.0932±0.0172Ad | 0.0027±0.0005Ad | 0.3680±0.0430Ab | 0.0094±0.0016Bc | 0.5918±0.0536Aa |
| Ethyl valerate (A51) | ZR | 0.0117±0.0026Bc | 0.0103±0.0043Cc | 0.1123±0.0944Aab | 0.0578±0.0091Bbc | 0.0396±0.0070Cbc | 0.1407±0.0152Ca |
|  | TR1-1-3 | 0.0376±0.0089Ac | 0.0210±0.0011Bc | 0.0319±0.0138Ac | 0.0287±0.0051Bc | 0.2194±0.0146Ab | 0.4119±0.0348Aa |
|  | ZF22 | ND | 0.0537±0.0046Ac | 0.0258±0.0020Ad | 0.3493±0.0130Aa | 0.0765±0.0084Bc | 0.1914±0.0291Bb |
| **Ketones** |  |  |  |  |  |  |  |
| 6-Dodecanone (A52) | ZR | ND | ND | 0.1533±0.0492Ba | 0.1096±0.0048Bb | 0.1583±0.0146Aa | ND |
|  | TR1-1-3 | ND | 0.1807±0.0085Ab | 0.2624±0.0419Aa | 0.236±0.0212Aa | ND | ND |
|  | ZF22 | ND | ND | 0.0890±0.0184Ba | ND | ND | ND |
| 2-Nonanone (A53) | ZR | ND | ND | 0.0308±0.0052Bd | 0.0380±0.0016Bc | 0.0527±0.0050Ab | 0.0602±0.0021Aa |
|  | TR1-1-3 | ND | 0.0265±0.0013Ac | 0.0863±0.0156Aa | ND | 0.0441±0.0037Bb | ND |
|  | ZF22 | ND | 0.0241±0.0026Ac | 0.0227±0.0027Bc | 0.0631±0.0020Aa | 0.0309±0.0015Cb | ND |
| 2-Heptanone (54) | ZR | ND | 0.0251±0.0092Ab | ND | ND | 0.0360±0.0007Aa | 0.0089±0.0010Ac |
|  | TR1-1-3 | ND | ND | 0.0468±0.0038Aa | 0.0356±0.0018Ab | ND | ND |
|  | ZF22 | ND | 0.0066±0.0006Bb | ND | 0.0077±0.0007Bb | 0.0269±0.0013Ba | ND |
| 6-Methyl-2-heptanone (A55) | ZR | ND | ND | 0.0140±0.0013Ba | 0.0097±0.0015Bb | 0.0161±0.0027Aa | ND |
|  | TR1-1-3 | ND | 0.0103±0.0005Ab | 0.0512±0.0164Aa | 0.0474±0.0005Aa | 0.0115±0.0004Bb | ND |
|  | ZF22 | 0.0112±0.0027Aa | ND | ND | ND | ND | ND |
| **Alkanes** |  |  |  |  |  |  |  |
| 1,1-Diethoxy ethane (A56) | ZR | ND | 0.1418±0.0799Aa | ND | 0.1281±0.014Aa | 0.1560±0.0104Aa | ND |
|  | TR1-1-3 | ND | 0.1342±0.0119Aa | 0.0941±0.0150Ab | 0.0899±0.0127Bb | 0.1029±0.0217Bb | ND |
|  | ZF22 | 0.3355±0.0385Aa | ND | ND | 0.0778±0.0056Bb | ND | 0.0886±0.0067Ab |
| 1,1, 3-Triethoxypropane (A57) | ZR | 0.0695±0.0156Ae | ND | 0.2379±0.0029Bc | 0.3661±0.0066Bb | 0.7248±0.0717Aa | 0.1667±0.0073Ad |
|  | TR1-1-3 | ND | 0.3994±0.0497Ab | 1.0385±0.2864Aa | 0.8489±0.0817Aa | 0.1037±0.0069Cc | 0.0993±0.0027Bc |
|  | ZF22 | ND | 0.2108±0.0255Bb | 0.3332±0.0408Ba | ND | 0.1843±0.0104Bb | 0.1049±0.0069Bc |

Supplementary TABLE 1 (*continued*)

| flavour compounds | group | 0d | 3d | 7d | 10d | 15d | 30d |
| --- | --- | --- | --- | --- | --- | --- | --- |
| 1,2-Epoxy-cycloheptane (A58) | ZR | 0.0045±0.0006Ba | 0.0024±0.0006Ab | 0.0042±0.0006Ba | 0.0040±0.0004Aa | ND | ND |
|  | TR1-1-3 | 0.0062±0.0010Ac | 0.0026±0.0001Acd | 0.0155±0.0043Aa | 0.0108±0.0023Ab | ND | 0.0053±0.0007Ac |
|  | ZF22 | 0.0049±0.0002Bb | 0.0032±0.0004Ac | 0.0029±0.0003Bc | 0.0069±0.0006Aa | ND | 0.0066±0.0013Aa |
| 2,3-Epoxyheptane (A59) | ZR | ND | ND | ND | ND | ND | ND |
|  | TR1-1-3 | 0.0167±0.0036Aa | ND | 0.0112±0.0018Ab | 0.0138±0.0004Aab | ND | ND |
|  | ZF22 | ND | ND | 0.0039±0.0006Bb | ND | ND | 0.0111±0.0005Aa |
| 2,6,10-Trimethyltetradecane (A60) | ZR | 0.0056±0.0011Bd | 0.0048±0.0019Bd | 0.0131±0.0022Bc | 0.0373±0.0047Bb | 0.0398±0.0033Ab | 0.0521±0.0074Aa |
|  | TR1-1-3 | 0.0304±0.0045Ab | 0.0194±0.0019Ac | 0.0472±0.0098Aa | 0.0344±0.0065Bb | 0.0368±0.0046Ab | 0.0294±0.0038Bbc |
|  | ZF22 | 0.0314±0.0058Ac | 0.0231±0.0052Ac | 0.0145±0.0024Bd | 0.0659±0.0057Aa | 0.0270±0.0013Cc | 0.0529±0.0043Ab |
| 6-Methyloctadecane (A61) | ZR | ND | ND | 0.0037±0.0016ABb | ND | 0.0071±0.0015ABa | ND |
|  | TR1-1-3 | ND | 0.0023±0.0002Ab | 0.0084±0.0017Aa | 0.0082±0.0058Aa | ND | ND |
|  | ZF22 | ND | ND | 0.0070±0.0036ABa | ND | 0.0110±0.0055Aa | ND |
| Nonane (A62) | ZR | ND | ND | 0.0164±0.0105Ba | 0.0062±0.0008Bb | ND | 0.0033±0.0001Ab |
|  | TR1-1-3 | ND | 0.0100±0.0014Ab | 0.0962±0.0230Aa | 0.0818±0.0063Aa | 0.0056±0.0011Bb | ND |
|  | ZF22 | ND | ND | ND | ND | ND | ND |
| 1,1-Diethoxypropane (A63) | ZR | 0.0067±0.0009Ac | 0.0448±0.0062Ab | ND | ND | ND | 0.2373±0.0101Ba |
|  | TR1-1-3 | 0.0101±0.0019Ab | ND | ND | ND | ND | 0.3467±0.0215Aa |
|  | ZF22 | 0.0106±0.0040Aa | 0.0443±0.0063Ab | ND | ND | ND | 0.2663±0.0177Ba |
| Amyl cyclopropane (A64) | ZR | ND | 0.0124±0.0051Ab | ND | 0.0142±0.0006Bb | ND | 0.0248±0.0105Aa |
|  | TR1-1-3 | ND | ND | ND | ND | 0.0102±0.0022Aa | ND |
|  | ZF22 | 0.0125±0.0034Ab | ND | ND | 0.0240±0.0002Aa | ND | ND |
| Trichloromethane (A65) | ZR | ND | 0.0198±0.0128Aa | ND | ND | ND | 0.0031±0.0001Bb |
|  | TR1-1-3 | 0.1643±0.0361Ab | ND | ND | ND | ND | 3.1718±0.2088Aa |
|  | ZF22 | 0.1517±0.0031Aa | 0.0266±0.0030Ab | ND | ND | ND | 0.0037±0.0009Bc |
| **Aromatic hydrocarbons** |  |  |  |  |  |  |  |
| p-Xylene (A66) | ZR | 0.0276±0.0029Ab | 0.0061±0.0025Ad | 0.0178±0.0096Ac | ND | ND | 0.0394±0.0032Ba |
|  | TR1-1-3 | 0.0092±0.006Ab | ND | ND | ND | ND | 0.0432±0.0049Ba |
|  | ZF22 | 0.0275±0.0041Ab | ND | ND | 0.0209±0.0021Ac | 0.0196±0.0003Ac | 0.0648±0.0054Aa |
| o-Xylene (A67) | ZR | 0.0155±0.0031Bb | 0.0091±0.0021Bc | ND | 0.0174±0.0011Bb | 0.0204±0.0004Ba | 0.0201±0.0015Ba |
|  | TR1-1-3 | 0.0292±0.0062Aa | 0.0116±0.0005Ab | 0.0195±0.0045Aa | 0.0225±0.0003Aa | 0.0232±0.0025Aa | 0.0198±0.0013Aa |
|  | ZF22 | 0.0196±0.0043Ba | 0.0155±0.0009Ab | 0.0183±0.0032Aa - | ND | ND | ND |
| o-isopropyl toluene (A68) |  | 0.0163±0.0107ABa | ND | 0.0151±0.0044Ba | 0.0111±0.0007Aa | ND | 0.0148±0.0039Aa |
|  | ZR | 0.0268±0.0187Aa | 0.0085±0.0025Aa | 0.0268±0.0056Aa | 0.020±0.01600Aa | 0.0109±0.0038Ba | 0.0102±0.0001Aa |
|  | TR1-1-3 | 0.0095±0.0032ABb | 0.0428±0.0387Aab | 0.0076±0.0017Bb | 0.0687±0.0568Aa | 0.0253±0.0129Aab | 0.0245±0.0215Aab |
| **Ene terpenoids** | ZF22 |  |  |  |  |  |  |
| 2 – pinene (A69) | ZR | ND | ND | 0.0040±0.0003Ac | 0.0062±0.0002Ab | 0.0084±0.0011Aa | ND |
|  | TR1-1-3 | ND | 0.0033±0.0005Bc | ND | 0.0054±0.0005Ab | 0.0071±0.0006Aa | ND |
|  | ZF22 | ND | 0.0054±0.0003Ab | ND | ND | 0.0078±0.0006Aa | ND |
| α- Caryophyllene (A70) | ZR | ND | 0.0037±0.0005Ae | 0.0044±0.0019Bcd | 0.0073±0.0009Abc | 0.0105±0.0016Ab | 0.0185±0.0038Aa |
|  | TR1-1-3 | 0.0103±0.0018Aa | 0.0033±0.0009Ab | 0.0100±0.0020Aa | ND | ND | ND |
|  | ZF22 | ND | ND | 0.0039±0.0003Bb | ND | 0.0074±0.0011Ba | ND |
| S - (-) – Limonene(A71) | ZR | ND | ND | 0.0243±0.0079Ab | 0.0262±0.0003Bb | ND | 0.0514±0.0019Aa |
|  | TR1-1-3 | ND | ND | ND | ND | 0.0254±0.0014Aa | ND |
|  | ZF22 | ND | 0.0210±0.0003Ac | ND | 0.0420±0.0017Aa | 0.0254±0.0021Ab | ND |
| δ – Elemene (A72) | ZR | 0.0073±0.0016Bd | 0.0045±0.0013Ae | 0.0085±0.0012Bcd | 0.0107±0.0009Ac | 0.0149±0.0015Ab | 0.0200±0.0014Ca |
|  | TR1-1-3 | 0.0125±0.006ABb | 0.0047±0.0004Ac | 0.0133±0.0029Ab | 0.0092±0.0008Abc | 0.0089±0.0003Cbc | 0.0327±0.0059Ba |
|  | ZF22 | 0.0097±0.0001ABcd | 0.0071±0.003Ad | 0.0069±0.0006Bd | 0.0167±0.0007Ab | 0.0127±0.001Bbc | 0.0853±0.0054Aa |

The results are expressed as the mean ± SD (n = 3). Capital letters indicate the same stage, and there is significant difference between different samples (*p* < 0.05); Lowercase letters indicate significant difference between different stages of the same sample (*p* < 0.05). A1-A72 is the number of volatile substances. ND indicates no detection.
